# Autonomic nervous system activity correlates with peak experiences induced by DMT and predicts increases in well-being

**DOI:** 10.1177/02698811241276788

**Published:** 2024-09-20

**Authors:** Valerie Bonnelle, Amanda Feilding, Fernando E Rosas, David J Nutt, Robin L Carhart-Harris, Christopher Timmermann

**Affiliations:** 1 The Beckley Foundation, Oxford, UK; 2 Division of Psychiatry, Department of Brain Sciences, Centre for Psychedelic Research, Imperial College London, London, UK; 3 Centre for Complexity Science, Imperial College London, London, UK; 4 Department of Informatics, University of Sussex, Brighton, UK; 5 Centre for Eudaimonia and Human Flourishing, University of Oxford, Oxford, UK; 6 Departments of Neurology and Psychiatry, University of California, San Francisco, San Francisco, CA, USA

**Keywords:** Autonomic nervous system, heart rate variability, peak experience, psychedelics, sympathovagal balance, sympathovagal coactivation

## Abstract

**Background:**

Non-ordinary states of consciousness induced by psychedelics can be accompanied by so-called “peak experiences,” characterized at the emotional level by their intensity and positive valence. These experiences are strong predictors of positive outcomes following psychedelic-assisted therapy, and it is therefore important to better understand their biology. Despite growing evidence that the autonomic nervous system (ANS) plays an important role in mediating emotional experiences, its involvement in the psychedelic experience is poorly understood. The aim of this study was to investigate to what extent changes in the relative influence of the sympathetic (SNS) and parasympathetic nervous systems (PNS) over cardiac activity may reflect the subjective experience induced by the short-acting psychedelic N,N-Dimethyltryptamine (DMT).

**Methods:**

We derived measures of SNS and PNS activity from the electrocardiograms of 17 participants (11 males, mean age = 33.8 years, SD = 8.3) while they received either DMT or placebo.

**Results:**

Results show that the joint influence of SNS and PNS (“sympathovagal coactivation”) over cardiac activity was positively related to participants’ ratings of “Spiritual Experience” and “Insightfulness” during the DMT experience, while also being related to improved well-being scores 2 weeks after the session. In addition, we found that the state of balance between the two ANS branches (“sympathovagal balance”) before DMT injection predicted scores of “Insightfulness” during the DMT experience, as well as subsequent sympathovagal coactivation.

**Conclusion:**

These findings demonstrate the involvement of the ANS in psychedelic-induced peak experiences and may pave the way to the development of biofeedback-based tools to enhance psychedelic therapy.

## Introduction

Psychedelics induce significant alterations in perceptual, cognitive, and emotional processing and, in scientific studies, can act as tools of perturbation for human consciousness (Timmermann et al., 2023a). Evidence suggests that intense positively valanced self-transcendent experiences (also called “peak experiences”) occurring during psychedelic therapy significantly predict positive mental health outcomes (reviewed in Ko et al., 2022), potentially mediating lasting symptom reductions in individuals suffering from treatment-resistant depression (Roseman et al., 2018), end-of-life existential distress (Bossis, 2021; Griffiths et al., 2016; Ross, 2018), or substance abuse disorders (Garcia-Romeu et al., 2015; Yaden and Griffiths, 2021). In healthy individuals, these peak experiences have also been associated with sudden, substantial, and sustained positive changes in behavior, personality and thought patterns, often precipitating a significant change in values and a new sense of purpose or meaning in life (Barrett and Griffiths, 2018; Bouso et al., 2018; Carhart-Harris et al., 2018; Erritzoe et al., 2018; Griffiths et al., 2006; McCulloch et al., 2022).

Despite the important therapeutic relevance of peak experiences, the mechanisms leading to these states remain unclear. In addition, even though great care may be taken to optimize psychedelic therapy to facilitate these experiences (Johnson et al., 2008), attempting to predict the affective features of a psychedelic experience is still challenging.

Contemporary research has largely focused on the effects of psychedelics on the central nervous system, often ignoring other biological mechanisms potentially involved in the emotional states induced by these substances. Yet, the brain and body are undeniably intrinsically and dynamically linked; perceptions, emotions, and cognition not only influence but also respond to the state of the body (Azzalini et al., 2019), as intuited by William James over a century ago (James, 1994) and emphasized by the embodied approach to cognitive neuroscience (Solms and Friston, 2018; Varela et al., 2017). The importance played by the autonomic nervous system (ANS)—particularly at the cardiac level—in emotional experiences and affective states is becoming increasingly recognized (Friedman, 2010; Kreibig, 2010; Pasquini et al., 2023). Although the centrality and specificity of the autonomic response are still subject to debate, recent work importantly demonstrated that cardiac sympathovagal activity may initiate emotional responses, preceding neural dynamics (Candia-Rivera et al., 2022). To what extent, then, are the intense emotional experiences fostered by psychedelics mediated by their impact on ANS activity and heart function?

The ANS has two main branches: the sympathetic nervous system (SNS) and the parasympathetic nervous system (PNS). The SNS is involved in priming the body and brain for action, triggering a series of physiological changes that are part of the stress response (“fight-or-flight”). The SNS has generally been proposed to be involved in emotional arousal when it is experienced with positive (e.g. joy, excitement) or negative (e.g. anxiety, hyper-arousal) valence (Kreibig, 2010; Laird, 2007). Classic psychedelics (e.g. LSD, psilocybin, mescaline, and N,N-dimethyltryptamine (DMT)) are serotonin-2A (5-HT2A) receptor agonists and exert effects consistent with activation of the SNS, particularly pupillary dilation, increases in heart rate and blood pressure, and increases in plasma stress hormones cortisol and epinephrine (Hasler et al., 2004; Olbrich et al., 2021; Passie et al., 2008; Strassman, 1996). The PNS on the other hand, is involved in rest and recovery after periods of stress, energy conservation/storage, and regulation of bodily functions (Karemaker, 2017). While parasympathetic activity has often been found to be associated with positive and pro-social emotions (Kok and Fredrickson, 2010; Kreibig, 2010), the relation between emotional valence and PNS is not straightforward (Kreibig, 2010). The PNS does not appear to be directly activated by classic psychedelics. Rather, serotonin 2A receptor antagonist ketanserin, known to reduce or even prevent psychoactive effects when administered simultaneously with a classic psychedelic, has been found to cause an increase in parasympathetic activity (Olbrich et al., 2021). The PNS might, however, become active throughout the experience, despite residual sympathetic activation, as a homeostatic response to the initial stress response induced by psychedelics, a mechanism known as “vagal rebound” (Laborde et al., 2018; Mezzacappa et al., 2001). The phase following the initial sympathetic activation by psychedelics may therefore be characterized by transient coactivation between SNS and PNS (Weissman and Mendes, 2021), a state we refer to as “sympathovagal coactivation.”

**Figure 1.**
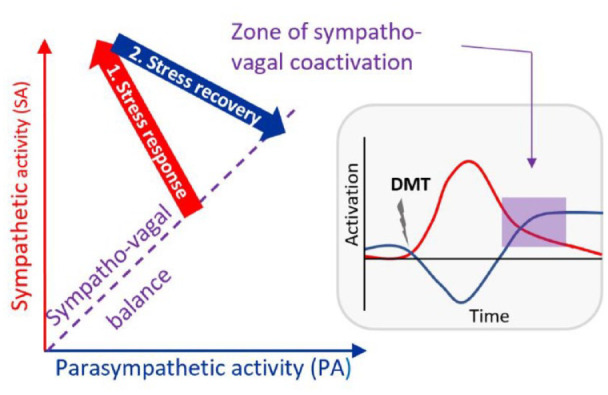
Proposed model for ANS involvement during the psychedelic experience. The hypothesis proposed here is that peak experiences, which are characterized by their intensity and positive balance, are associated with the dual influence of the sympathetic (red) and parasympathetic branches of the ANS (blue) over cardiac activity. Psychedelics may facilitate the induction of this “sympathovagal coactivation” by triggering an initial stress response associated with strong sympathetic stimulation and moderate parasympathetic withdrawal, followed by a recovery phase largely mediated by parasympathetic activity increase. We further hypothesized that starting the experience from a state of balance between the SNS and PNS (sympathovagal balance) may be optimal in order to return to a balanced, but more activated state, after the initial stress response, which we hypothesize to be optimal for peak experiences. ANS: autonomic nervous system.

Intensely pleasurable experiences are often associated with autonomic manifestations (e.g. “chills”), which are accompanied by physiological markers of arousal such as increased heart rate (Blood and Zatorre, 2001). On the other hand, many types of contemplative practices (e.g. mindfulness meditation), which can also lead to peak experiences, are typically associated with increased parasympathetic activity (Fiorentini et al., 2013; Gerritsen and Band, 2018). However, to our knowledge, the interplay between autonomic activity and peak experiences—which share some characteristics of both intensely pleasurable and contemplative experiences—has never been investigated. Here, *we hypothesized that the state of sympatho-vagal coactivation that may follow the intense initial stress response induced by psychedelics could be conducive of peak experiences, which are characterized by their intensity/high arousal (SNS activity) and contemplative quality (PNS activity)* (Figure 1).

Furthermore, if we consider sympathetic tone as reflecting our ability to respond to stress (and/or salience), and parasympathetic tone as our ability to regulate and restore homeostasis after a stress response, it then becomes apparent that a state of balance between the two branches (“sympathovagal balance”) can facilitate the transition between high and low arousal states, rapidly modulating the physiological and emotional arousal elicited by environmental stressors, which might be beneficial for adaptation, resilience to stress, and emotional flexibility (An et al., 2020; Appelhans and Luecken, 2006; Kok and Fredrickson, 2010). These attributes may play a crucial role in navigating the often challenging emotional states induced by psychedelics (Campo, 2022), and promote an effective post-stress recovery response, which as we hypothesized, might be associated with favorable psychedelic experiences. *We therefore propose a second hypothesis whereby starting the psychedelic experience from a state of greater sympatho-vagal balance may facilitate the occurrence of peak experiences* (Figure 1).

DMT, a short-acting serotonergic psychedelic with therapeutic potential (D’Souza et al., 2022; Timmermann et al., 2024) offers a unique opportunity to investigate shifts in autonomic activity and their relation to the psychedelic experience as its short duration of action (~10–15 min) allows drawing more accurate relationships between subjective reports of acute effects, physiological measures, and mental health outcomes. Crucially, it also provides an optimal opportunity to assess how these dynamics relate to peak experiences, and changes in well-being. Here, we used a set of electrocardiograms (ECG) data collected as part of a neuroimaging study of the DMT experience (Timmermann et al., 2023b) to conduct (1) an exploratory post hoc analysis of the complete profiles of SNS and PNS fluctuations throughout the DMT experience, and (2) an ad hoc analysis focusing on a specific set of hypotheses introduced above regarding the involvement of sympathovagal coactivation and balance in peak experiences.

## Materials and methods

### Participants and experimental procedures

The original study, from which the data used for the analyses presented in this article stem, was designed as a single-blinded placebo-controlled trial in healthy participants. Experimental sessions consisted of continuous and simultaneous functional magnetic resonance imaging – electroencephalogram (fMRI-EEG) resting-state scans, with ECG recording, which lasted 28 min, with DMT or placebo administered at the end of the eighth minute. In total, 20 participants completed all study visits, but only 17 (11 males, mean age = 33.8 years, SD = 8.3) had reliable ECG data (i.e. less than 10% noisy segments). Further details on the study design can be found elsewhere (Timmermann et al., 2023b), and in Supplemental Information (SI).

This study was approved by the National Research Ethics Committee London—Brent and the Health Research Authority and was conducted under the guidelines of the revised Declaration of Helsinki (2000), the International Committee on Harmonization Good Clinical Practices guidelines, and the National Health Service Research Governance Framework. Imperial College London sponsored the research, which was conducted under a Home Office license for research with Schedule 1 drugs.

### Subjective ratings

Following the approach in Olbrich et al. (2021), we used the scores on the 11 Dimensions Altered States of Consciousness Questionnaire—11D-ASC (Studerus et al., 2010) to characterize participants’ subjective experience during their DMT session. The 11D-ASC features the following subscales: *Experience of Unity*, *Spiritual Experience*, *Blissful State*, and *Insightfulness Impaired Control and Cognition*, *Anxiety Disembodiment*, *Complex Imagery*, *Elementary imagery*, *Synesthesia* and *Meaning*. Although we were particularly interested in the subscales relevant to the peak experience, all the subscales were included in the analysis to control for the specificity of the effects observed. Participants also completed the Well-Being Index questionnaire (5-item World Health Organization Well-Being Index - WHO-5) WHO-5) at baseline and 2 weeks after their last DMT session (Topp et al., 2015).

### ECG recording

#### Data collection

ECG data was collected using two electrodes. One was placed on participants’ backs (behind the chest area), and the other was placed above the heart area. The data was recorded with an MR-compatible BrainAmp MR amplifier (BrainProducts GmbH, Munich, Germany). The data was recorded with a sampling rate was 5 kHz, and with a hardware 250 Hz low-pass filter. Recordings lasted 28 min, with 8 min of baseline and 20 min post-injection.

#### Pre-processing

ECG data was demeaned and band-passed filtered at 1-30 Hz. It was then exported in text format to Kubios Scientific software (Tarvainen et al., 2014). Automatic R-peak detection was applied, and all identified R-peaks were again manually inspected. Where necessary, R-peaks were corrected manually. Data sets with >10% of noisy segments (i.e. without the possibility of reliable R-peak detection due to artifacts) were discarded from further analysis. Three participants for the DMT session and four participants—same three plus another—for the placebo session, were discarded due to this procedure.

### Measures of ANS activity

#### SNS and PNS indexes computation

**Figure 2.**
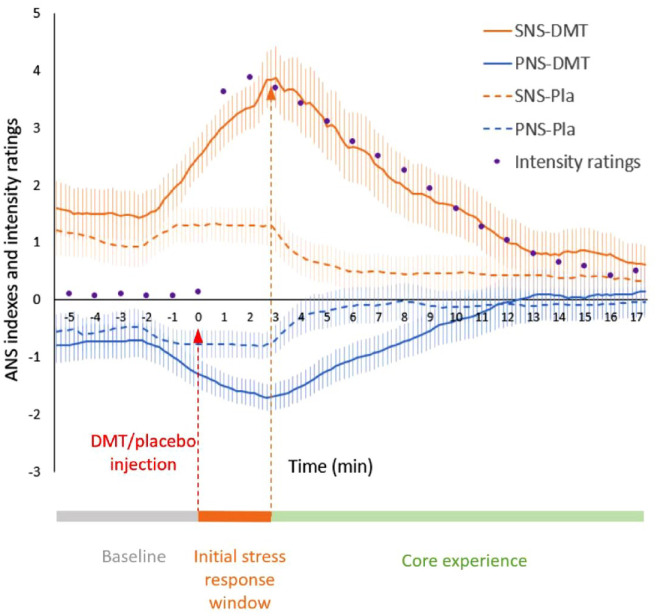
Measures of SNS (orange) and PNS (blue) activity, estimated based on 180 s of heart rate variability (HRV) data, with 20 s increments, were averaged across the 17 participants, for the DMT (continuous lines) and the placebo sessions (dotted line). Error bars indicate standard errors. Intensity ratings (purple dots), corrected within the same participants but during a distinct DMT session, were averaged across participants and normalized to match the levels of SNS indexes for visualization purposes. Significant differences between DMT and placebo can be found in Supplemental Table S1. The initial stress response, window was defined as the phase during which SNS ascents. Note that for both DMT and placebo, SNS starts increasing before the injection, most likely due to anticipatory processes. DMT: N,N-dimethyltryptamine; PNS: parasympathetic nervous system; SNS: sympathetic nervous system.

A similar procedure to that used in Olbrich et al. (2021) was used, whereby indexes of SNS and PNS activity were computed in the Kubios software, based on a proprietary combination of time-domain measures (for PNS: mean RR intervals and root mean square of successive differences between normal heartbeats; for SNS: mean heart rate and Baevski Stress Index) and nonlinear measures (standard deviations SD1 and SD2 derived from the Poincaré plot of RR time series, with SD1 reflecting vagally mediated short-term RR-intervals variability, and SD2 reflecting sympathetically/stress hormones-mediated long-term RR variability) (see SI). These indexes provide reliable estimates of the ANS activity compared to normal resting values (Olbrich et al., 2021). For the estimation of SNS and PNS time courses, the indexes were computed based on 180 s windows, with 60 s steps. The last indexes were therefore computed at 25:30 min. For the analyses relating to specific periods (i.e. baseline or core experiences), the indexes were computed based on the corresponding section of RR interval data (3–7 min for baseline, 11–25 min for core experience, see Figure 2).

#### Measures of sympathovagal balance

There is some controversy around the use of the ratio of low-frequency to high-frequency power—computed from Fast Fourier Transformation of R-R peaks time series—as an index of sympathovagal balance (Billman, 2013; Shaffer et al., 2014). Instead, we used the ratio of Poincaré plot indexes SD1 to SD2, SD1/SD2 (see SI Methods) (Shaffer and Ginsberg, 2017).

#### Measure of sympathovagal coactivation

According to our hypothesis, peak experiences would be associated with a state of coactivation of SNS and PNS. The interaction term SNS × PNS was computed to reflect this dual influence, after having translated both indexes into a range of strictly positive values, and was subsequently used as an additional variable for the linear regressions, as well as for the correlations with subjective measures.

### Statistical analysis

To visualize the time course of SNS and PNS activity during the DMT and placebo sessions (Figure 2), the indexes for each time point were averaged across participants. Paired samples *t*-tests were used to identify the time points where SNS and PNS indexes were significantly different between the DMT and placebo sessions (Supplemental Table S1).

A linear regression analysis approach was used to evaluate the respective influence of SNS and PNS activity during the core DMT experience (i.e. after the initial stress response) on each dimension of the 11D-ASC questionnaire. One of our hypotheses being that coactivation of SNS and PNS plays an important part in the peak experience, we therefore added an interaction term to the model (SNS × PNS), reflecting the dual influence of the two branches over cardiac activity. Multicollinearity was assessed in SPSS with the collinearity diagnostic variables Variance Inflation Factors (VIF) and tolerance threshold (T). SNS and PNS’s collinearity was moderate but within an acceptable range, that is VIF < 5 and T > 0.2 (SNS: VIF = 3.213, T = 0.311; PNS: VIF = 3.274, T = 0.305), and low for the interaction term SNS × PNS (VIF = 1.155, T = 0.866) (Marcoulides and Raykov, 2019).

Spearman correlations between minute-by-minute ANS indexes and subjective ratings were also performed to identify the periods of the experience during which the relation between ANS and subjective experience was strongest.

Finally, participants were split into two groups based on the change in well-being reports from baseline to follow-up assessment at 2 weeks (WHO-5). This procedure was used to account for the small variance in the well-being reports scores, and participants within- and inter-individual variabilities in estimating and reporting their well-being scores on two occasions. Eight participants showed an improvement in their well-being scores (change in well-being scores > 0, group average = 1.625), eight showed no improvement, or a reduction (change in well-being ⩽ 0, group average = −1.5), and well-being data was missing for one participant. SNS × PNS scores during the core experience were compared between the two groups using an independent samples *t*-test.

## Results

### Effect of DMT versus placebo on SNS and PNS activity profiles

The time courses of SNS and PNS activity, which were derived from ECG data collected during an 8 min baseline period and for 20 min post-DMT/placebo injection (see section “Materials and Methods”), were averaged across participants during the DMT and the placebo session and plotted on Figure 2, along with during-experience subjective intensity ratings. From the time of injection, SNS activity increased, and PNS activity decreased significantly in the DMT condition compared to placebo. SNS remained significantly higher in the DMT condition compared to placebo for 11 min post-injection, and PNS remained significantly lower for 8 min (see Supplemental Table S1).

### Association between ANS measures during the DMT experience and subjective experience ratings

Results from the linear regressions, shown in Table 1, indicate that the interaction term between SNS and PNS during the core DMT experience (i.e. after the initial stress response) is a significant positive factor influencing subjective reports of *Spiritual Experience* (β = 0.83, *p* = 0.023) and *Insightfulness* (β = 0.684, *p* = 0.007). This is in keeping with our hypothesis of the dual involvement of the SNS and PNS (i.e. sympathovagal coactivation) in the peak experience. SNS activity during the DMT experience also appears to be negatively related to subjective ratings of *Impaired Control and Cognition* (β = −0.959, *p* = 0.019).

**Table 1.** Standardized coefficients and associated *p* values for linear regressions assessing the influence of SNS, PNS, and the interaction term SNS × PNS (sympathovagal coactivation) during the core experience on the subscales of the 11D-ASC questionnaire.

11D-ASC dimension	SNS	PNS	SNS × PNS
Unity	0.667, *p* = 0.112	0.119, *p* = 0.789	0.167, *p* = 0.489
Spiritual experience	0.077, *p* = 0.844	0.164, *p* = 0.680	0.83,* *p* = 0.023
Blissful state	−0.048, *p* = 0.912	−0.022, *p* = 0.960	0.522, *p* = 0.062
Insightfulness	0.091, *p* = 0.803	0.009, *p* = 0.980	0.684,** *p* = 0.007
Disembodiment	−0.339, *p* = 0.482	−0.174, *p* = 0.719	−0.201, *p* = 0.488
Impaired cognition	−0.959,* *p* = 0.019	−0.472, *p* = 0.213	−0.159, *p* = 0.470
Anxiety	−0.654, *p* = 0.139	−0.189, *p* = −0.186	−0.134, *p* = 0.599
Complex imagery	−0.105, *p* = 0.832	−0.026, *p* = 0.958	0.204, *p* = 0.496
Elementary imagery	0.326, *p* = 0.457	−0.173, *p* = 0.693	−0.196, *p* = 0.457
Synaesthesia	−0.650, *p* = 0.183	−0.575, *p* = 0.240	0.193, *p* = 0.499
Meaning	−0.420, *p* = 0.386	−0.180, *p* = 0.709	−0.123, *p* = 0.669

11D-ASC: 11 Dimensions Altered States of Consciousness Questionnaire; PNS: parasympathetic nervous system; SNS: sympathetic nervous system.

* *p* < 0.05. **p < 0.01.

To explore in more detail which phases of the experience showed the strongest correlations between subjective ratings and SNS × PNS measures, Spearman correlations were performed between the 11 subscales of the 11D-ASC and minute-by-minute fluctuations of the SNS × PNS indices, calculated based on 180 s RR-intervals data (Supplemental Table S2). SNS × PNS was significantly and positively correlated with *Spiritual Experience from* minute 4–13 post-injection, and with *Insightfulness*, with correlations starting at baseline (minutes −5 to −3, maximum at minute −3), and resurfacing later in the experience at minutes 12 and 13 (maximum at minute 13, Supplemental Table S2).

### Impact of baseline autonomic balance on the quality of the peak experience

We hypothesized that peak experiences would be facilitated when the two branches of the ANS are well balanced at baseline, which would facilitate the engagement of the SNS as part of the stress response followed by the vagally mediated stress response regulation. We, therefore, investigated the impact of autonomic balance at baseline on subjective ratings, using an established index of sympathovagal balance derived from the Poincaré plot of the R-R intervals, SD1/SD2 (Shaffer and Ginsberg, 2017). SD1/SD2 at baseline significantly related to *Spiritual Experience* ratings (*n* = 17, *r* = 0.555, *p* = 0.021), and related marginally to *Insightfulness* (*n* = 17, *r* = 0.413, *p* = 0.099), but not to any other ratings (Supplemental Table S3). Individuals who entered the DMT experience with an SD1/SD2 score closer to 1 (i.e. with a more balanced ANS), scored higher on the *Spiritual Experience* subscale of the 11D-ASC (Supplemental Figure S1). SD1/SD2 was also correlated with subsequent SNS × PNS coactivation during the DMT experience (*n* = 17, *r* = 0.484, *p* = 0.049), and the absence of a significant partial correlation between SD1/SD2 and *Spiritual Experience* ratings, when controlling for sympathovagal coactivation (df = 14, *r* = 0.175, *p* = 0.516), suggests that coactivation during the DMT experience may mediate this predictive relationship between balance and *Spiritual Experience.*

### Changes in ANS during the experience are related to long-term changes in well-being

Participants were split into two groups based on whether they had reported an improvement in their well-being in the 2 weeks following their DMT experience. Group 1 reported no change or a reduction in well-being (*n* = 8, mean change in WHO-5 from baseline: -1.50 ± 1.6 SD). Group 2 reported improved well-being relative to baseline (*n* = 8, mean change in WHO-5 from baseline: 1.62 ± 0.91). In terms of quality of the DMT experience, Group 2 showed significantly higher ratings of *Blissful Experience* compared to Group 1 (*t* = 2.7, df = 14, *p* = 0.017), with no other significant differences for the other subscales of the 11D-ASC. Importantly, when comparing ANS activity between the two groups, Group 2 (improved well-being) showed a higher SNS × PNS coactivation index during the DMT experience compared with Group 1 (*t* = 2.32, df = 14, *p* = 0.036), but no significant difference in SNS or PNS activity alone.

## Discussion

The aim of this study was to explore whether, and to what extent, changes in autonomic function during DMT administration related to the content of subjective experiences—as well as later changes in well-being. Our hypotheses were that positively experienced peak states would be associated with the coactivation of sympathetic and parasympathetic autonomic branches. We also hypothesized that entering the experience from a state of greater sympathovagal balance would facilitate a better engagement of the two branches during the experience, thereby favoring the occurrence of peak experiences.

The present findings confirmed our hypothesis of the dual involvement of PNS and SNS in peak experiences. More specifically, we found these effects to be more pronounced in the *Spiritual Experience* and *Insightfulness* subscales, both positive features of peak states induced by psychedelics (Studerus et al., 2010). Importantly, sympathovagal coactivation during the experience was a better predictor of subsequent improvement in well-being than subjective reports. This state of coactivation could be the result of cardiovascular recovery from physiological stress, a process known as “vagal rebound” associated with increased vagal modulation, despite residual sympathetic activation (Mezzacappa et al., 2001). Recovery from a stressor has indeed been shown to be associated with transient SNS and PNS coactivation (Weissman and Mendes, 2021). Vagal rebound has been associated with the release of oxytocin and brain-derived neurotropic factor (BDNF), both of which have been associated with faster vagal recovery and cortisol level reduction after stress (Engert et al., 2016; Linz et al., 2019), and can also be found in blood plasma following psychedelic experiences (Holze et al., 2022; Hutten et al., 2020). Beta-endorphins are also released in the stress recovery phase (Pilozzi et al., 2020). In this state, the SNS may continue promoting the deployment of metabolic energy resources, which is associated with an increase in bottom-up attentional processing (Sänger et al., 2014), and a reduction of power in alpha brainwaves (Kim et al., 2021; Schubring and Schupp, 2019)—a finding consistently found with psychedelics, including DMT (Pallavicini et al., 2021; Timmermann et al., 2019), may result in an enrichment of the field of sensory experience. Simultaneously, PNS activity and the associated stress recovery hormones, emerging after the initial stress response, may give the experience a particularly positive and soothing taint, promoting a state of “peaceful arousal.” Future work should evaluate how long this vagal rebound may last if it is indeed associated with increased BDNF, oxytocin, and beta-endorphins, and to what extent it may underlie the frequently occurring post-psychedelic “afterglow” phase (Evens et al., 2023).

Future work should also investigate whether the present findings are specific to peak experiences induced by DMT or if they can be extended to other psychedelics and non-drug-induced non-ordinary states of consciousness (NSCs). Such a physiological marker of peak experience would allow the implementation of techniques aimed at guiding individuals undergoing psychedelic-assisted therapy toward favorable physiological states via biofeedback, where behavior (e.g. breathing) is guided by real-time feedback of the ANS. Furthermore, these physiological markers of peak states could serve as objective measures of the quality of the psychedelic experience, which could be particularly valuable given the ineffability inherent to peak experiences. It, however, remains to be seen whether peak experiences always emerge from this sympathovagal coactivation state, or if it simply constitutes a favorable condition for their emergence.

Another study examining the effects of several psychedelics on cardiac activity reported consistent increases in heart rate (reflecting elevated SNS activity) and high-frequency heart rate variability (HF-HRV) (reflecting elevated PNS activity), as well as increased heart rate entropy during the psychedelic experience (Rosas et al., 2023). It is tempting to propose that heart rate entropy may be related to the dual influence of SNS and PNS over cardiac activity, as the contradicting excitatory and inhibitory impact of the two branches may enhance the complexity of the resulting cardiac activity pattern. Indeed, the Poincaré plot-derived measure, SD1/SD2, which represents the ratio between short R-R interval variation and long interval variation, has been described both as a measure of sympathovagal balance, and a measure of complexity of the HR signal (Pham et al., 2021; Shaffer and Ginsberg, 2017). Importantly, in Rosas et al.’s study, changes in heart rate entropy were correlated with changes in brain entropy in multiple brain regions involved, among other things, in self-referential processes and metacognition (anterior and posterior cingulate cortex, precuneus), perception of emotions and social cognition (superior temporal gyrus), self-transcendence and interpretation of sensory information (left inferior parietal lobe), as well as sensorimotor areas. Increased brain entropy under psychedelics, a state associated with an expansion of the repertoire of active brain states (Atasoy et al., 2017), has been proposed to relate to psychological flexibility and insight and has been found to reflect the richness of subjective experience, possibly via the relaxation of prior beliefs and expectations (Carhart-Harris and Friston, 2019; Carhart-Harris et al., 2014). A possible mechanism, which will have to be tested in future studies, therefore emerges, whereby SNS and PNS’s dual influence over cardiac activity may not only generate higher heart rate entropy, but also promote a state of higher brain entropy via afferent communication pathways, associated with a richer state of consciousness, and greater flexibility and the development of novel insights.

Of interest, increased spirituality and religiosity have been observed in individuals with brain lesions to the periaqueductal gray, a region that promotes sympathetic activation while suppressing vagal activity (Larkin et al., 2021; Nosaka et al., 1996), and that plays a critical role in triggering defensive behaviors, including fear, panic attacks, and anxiety, in response to threats (Ferguson et al., 2022). Lesions to this area may prevent individuals from experiencing stress-related SNS activation as aversive or anxiogenic, and, by suppressing (or reducing) the disengagement of the PNS in response to a stressor, may facilitate the co-deployment of the two ANS branches.

It is important to note that, although the present results confirm our hypothesis that sympathovagal coactivation is an optimal physiological state for the occurrence of psychedelic-induced peak experience, SNS and PNS can also coactivate under other circumstances that are not related to such experiences, such as the “freezing” state (Roelofs, 2017), or certain forms of panic disorder, whereby increased excitability of both the PNS and SNS, referred to as amphotonia, can occur (Nutt, 1989). Possibly accounting for this apparent discrepancy, according to a popular theory, the PNS may be further divided into two sub-systems—a ventral one associated with rest, safety, and pro-social behaviors, and a dorsal one associated with immobilization behaviors (Porges, 2007). Only coactivation of the SNS with the ventral branch of the PNS may promote the occurrence of peak experiences.

The present results also confirmed our second hypothesis that greater sympathovagal balance at baseline would be related to the quality of the psychedelic experience, by allowing an optimal engagement of the SNS while also promoting an efficient post-stress recovery vagal activation. Indeed, high vagal tone (i.e. PNS activity) may hinder the intensity and duration of the stress response, and it has been proposed that lower baseline vagal tone and high SNS activity may index greater preparedness to engage a stress response (Matsumura et al., 2021), whereas higher baseline vagal tone may reflect a propensity to evaluate safety in the environment (Miller et al., 2017; Porges, 2022; Thayer et al., 2012). On the other hand, high sympathetic activity has been associated with vulnerability to stress and impaired emotional regulation, reflecting less effective PNS-mediated post-stress recovery mechanisms (Appelhans and Luecken, 2006; Pinna and Edwards, 2020). Starting the psychedelic experience from a state of balance between the two branches would therefore promote the deployment of optimal sympathetic stress response and post-stress vagal rebound. We found that baseline SD1/SD2 could predict spiritual experience ratings and that this relationship was mediated by sympathovagal coactivation during the DMT experience. Sympathovagal balance may therefore provide a physiological marker of “readiness” to undergo a deeper and more transformative psychedelic experience, characterized by strong SNS activation and subsequent vagal rebound. This finding is consistent with the notion, pursued in some contemplative practices such as Zen Buddhism, that finding the right equilibrium between arousal and rest, tension and relaxation, is key to the meditative practice (*Gudo Nishima, Zen Master Gudo Nishijima On Zen & The Nervous System 4Min*, 2015). If indeed meditation practices allow the cultivation of a more balanced ANS, as suggested in a study that identified increase sympathovagal balance after an advanced meditation program (Kacker et al., 2016), our findings may provide mechanistic evidence for previous observations that contemplative practices may aid in the preparation for psychedelic experiences to foster safety and positive effects (Smigielski et al., 2019; Timmermann et al., 2023a). Our findings may thus have translational relevance by suggesting that cardio-physiological state may be a key factor for the success of the clinical use of psychedelics, complementing preliminary findings that psychological preparedness may promote the safety and efficacy of psychedelic therapy (Aday et al., 2021). Furthermore, our findings are in line with suggestions that the cultivation of optimal psychological and physiological states can improve safety and efficacy of psychedelics and NSCs, more broadly (Timmermann et al., 2022; Timmermann et al., 2023a). These results warrant further research into the potential to develop markers of “readiness for acute alterations of consciousness” based on the state of the ANS, which could be used as predictors of peak experiences, and thereby of positive outcomes in psychedelic-assisted therapy (Roseman et al., 2018; Yaden and Griffiths, 2021).

DMT induced a pronounced increase in SNS activity, with a profile that appeared to directly parallel that of subjective intensity ratings (Figure 2). Though this association could simply relate to greater target receptor engagement by DMT, other explanations are possible. Indeed, other forms of NSCs are also known to be associated with, and even induced by, states of high arousal. For instance, direct manipulation of the SNS through specific breathing practices (i.e. hyperventilation) can induce NSCs similar to those induced by psychedelics (Davidson, 1976; Fincham et al., 2023; Oswald et al., 2023). Noteworthily, near-death experiences (NDEs), which are often described as mystical states that share some commonalities with DMT (Timmermann et al., 2018) and 5-MeO-DMT experiences (Michael et al., 2023), and have been shown to entail states of “hyper-lucidity” or psychological clarity (Cassol et al., 2018), are associated with intense sympathetic activation caused by physiological stress such as hypoxia (Xu et al., 2023). If NDEs are often described as promoting long-term beneficial changes in attitudes, beliefs, and values, they may also lead to symptoms of posttraumatic stress disorder (Greyson, 2013). The Pivotal Mental States theory proposes that periods of intense acute stress promote a hyper-plastic state able “to kindle conditions for major, potentially lasting, psychological change, pivotable either towards illness or wellness” (Brouwer and Carhart-Harris, 2021). It would be tempting to propose that the engagement of post-stress recovery vagal activity may significantly weigh on the bifurcation toward positive transformation or trauma.

The analytical approach used in this study was partly inspired by that of Olbrich et al. (2021). In their study, they found that LSD caused an increase in SNS activity and a decrease in PNS activity, which were respectively positively and negatively associated with most scales of the 11D-ASC, whether of positive (e.g. *Spiritual Experience*) or negative (e.g. *Anxiety*) valence (Olbrich et al., 2021). While their findings are consistent with our observation that SNS activity appears to be closely related to the general intensity of the experience, SNS, we found, was negatively related to challenging experience elements such as Impaired Control and Cognition. If these differences may indicate different autonomic effects of LSD and DMT, it should also be noted that, in Olbrich’s study, cardiac activity was only measured during two relatively small time windows, out of many hours of LSD-induced psychedelic experience, making it difficult to relate ECG-derived markers of sympathovagal activity to the overall subjective experience ratings collected at the end of the session. In our study, the use of the short-acting compound DMT represents a significant advantage as we could track the profile of SNS and PNS activity over the course of the entire experience, and relate the average measures during the core DMT experience with subjective ratings. Furthermore, the availability of ECG measurements at baseline, before DMT intake, allowed the assessment of the relevance of ANS measures in predicting the quality of the subsequent psychedelic experience.

Although we were able to identify clear relationships between acute changes in ANS and subjective experiences elicited by DMT, the question of whether changes in the ANS are driving or following changes at the level of the central nervous system remains open. This could be addressed in future studies by comparing the time courses of ANS fluctuations with that of brain activity determined via electroencephalography or functional magnetic resonance imaging, in an approach similar to that of Candia-Rivera et al. (2022). While we did not address causality between the ANS and the central nervous system, we did find a prediction of positive peak experiences via baseline ANS activity, which suggests a causal link from the ANS to phenomenology. Future studies should also aim to complement measures of autonomic activity derived from cardiac activity with other autonomic measures such as electrodermal activity, pupillometry, or piloerection.

The present results support the hypothesis that the remarkable psychological effects of psychedelics are not only the result of cortical neuronal mechanisms but involve multiple bodily systems—including the ANS. Specifically, we found that sympathovagal balance and coactivation, are key aspects of both peak experiences and improved well-being. These findings may help the development of biofeedback techniques to guide the ANS toward states that are conducive to experiences that facilitate better mental health outcomes.

## Supplemental Material

sj-docx-1-jop-10.1177_02698811241276788 – Supplemental material for Autonomic nervous system activity correlates with peak experiences induced by DMT and predicts increases in well-being Supplemental material, sj-docx-1-jop-10.1177_02698811241276788 for Autonomic nervous system activity correlates with peak experiences induced by DMT and predicts increases in well-being by Valerie Bonnelle, Amanda Feilding, Fernando E Rosas, David J Nutt, Robin L Carhart-Harris and Christopher Timmermann in Journal of Psychopharmacology
