# Supplementary material for: Autonomic nervous system activity correlates with peak experiences induced by DMT and predicts increases in well-being
Source: J Psychopharmacol. 2024 Sep 20;38(10):887–96. doi: 10.1177/02698811241276788 (PMC11512487; doi:10.1177/02698811241276788)
Supplement: sj-docx-1-jop-10.1177_02698811241276788 – Supplemental material for Autonomic nervous system activity correlates with peak experiences induced by DMT and predicts increases in well-being [file sj-docx-1-jop-10.1177_02698811241276788.docx]

**SUPPLEMENTARY INFORMATION (SI)**

SI TABLES

**Table S1**

P values for the paired sample T-test comparing SNS, PNS and intensity measures between DMT and placebo minute by minute

| Time relative to injection (min) | SNS | PNS | Intensity ratings |
| --- | --- | --- | --- |
| -5 | 0.818 | 0.764 | 0.083 |
| -4 | 0.587 | 0.852 | 0.301 |
| -3 | 0.417 | 0.726 | 0.332 |
| -2 | 0.495 | 0.584 | 0.163 |
| -1 | 0.115 | 0.205 | 0.332 |
| 0 | 0.001 | 0.032 | 0.104 |
| 1 | <0.001 | 0.005 | <0.001 |
| 2 | <0.001 | 0.003 | <0.001 |
| 3 | <0.001 | 0.002 | <0.001 |
| 4 | <0.001 | 0.004 | <0.001 |
| 5 | <0.001 | 0.006 | <0.001 |
| 6 | <0.001 | 0.008 | <0.001 |
| 7 | 0.002 | 0.020 | <0.001 |
| 8 | 0.002 | 0.039 | <0.001 |
| 9 | 0.003 | 0.355 | <0.001 |
| 10 | 0.006 | 0.634 | <0.001 |
| 11 | 0.029 | 0.926 | <0.001 |
| 12 | 0.127 | 0.737 | 0.003 |
| 13 | 0.178 | 0.540 | 0.002 |
| 14 | 0.099 | 0.620 | 0.004 |
| 15 | 0.096 | 0.544 | 0.003 |
| 16 | 0.126 | 0.546 | 0.007 |
| 17 | 0.195 | 0.527 | 0.026 |

**Table S2**

Correlations SNSxPNS minute by minute and subjective experience scores (11D-ASC). *indicates p<0.05 and p<0.01 respectively. Only r values for which p<0.1 are shown.

| Time relative to injection (min) | Unity | Spiritual Experience | Blissful state | Insight. | Disembod. | Impaired Cognition | Anxiety | Complex Imagery | Elementary Imagery | Synaesthesia | Meaning |
| --- | --- | --- | --- | --- | --- | --- | --- | --- | --- | --- | --- |
| -5 |  |  |  | .556* |  |  |  |  |  |  |  |
| -4 |  | .424 |  | .556* |  |  |  |  |  |  |  |
| -3 |  | .437 |  | .568* |  |  |  |  |  |  |  |
| -2 |  |  |  |  |  |  |  |  |  |  |  |
| -1 |  |  |  |  |  |  |  |  |  |  |  |
| 0 |  |  |  |  |  |  |  |  |  |  |  |
| 1 |  | .433 |  |  |  |  |  |  |  |  |  |
| 2 |  | .451 |  |  |  |  |  |  |  |  |  |
| 3 |  | .424 |  |  |  |  |  |  |  |  |  |
| 4 |  | .535* |  |  |  |  |  |  |  |  |  |
| 5 |  | .475 |  |  |  |  |  |  |  |  |  |
| 6 |  | .482* |  |  |  |  |  |  |  |  |  |
| 7 |  | .536* |  |  |  |  |  |  |  |  |  |
| 8 |  | .563* |  | .437 |  |  |  |  |  |  |  |
| 9 |  | .450 |  |  |  |  |  |  |  |  |  |
| 10 |  | .531* |  | .429 |  |  |  |  |  |  |  |
| 11 |  | .543* |  | .470 |  |  |  |  |  |  |  |
| 12 |  | .587* |  | .503* |  |  |  |  |  |  |  |
| 13 |  | .522* |  | .531* |  |  |  |  |  |  |  |
| 14 |  | .477 |  | .475 |  |  |  |  |  |  |  |
| 15 |  | .450 |  | .455 |  |  |  |  |  |  |  |
| 16 |  | .428 |  |  |  |  |  |  | -.477 |  |  |
| 17 |  | .451 |  |  |  |  |  |  | -.450 |  |  |

**Table S3:**  Correlation between measures of sympathovagal (i.e., SNS-PNS) balance at baseline and during core experience and subjective ratings of the quality of the peak experience. * indicates p<0.05

|  | **SD1/SD2 Baseline** |
| --- | --- |
| **Experience of Unity** | r=-.097 |
| **Spiritual Experience** | r=.555* |
| **Blissful state** | r=.126 |
| **Insightfulness** | r=.413 |
| **Impaired Cog.** | r=-.177 |
| **Anxiety** | r=-.190 |

SI FIGURE

**Figure S1:** **Correlation between autonomic balance at baseline and Spiritual Experience ratings**

The x axis corresponds to the ratio of SD1 to SD2, measures derived from the Poincaré plot of the RR-intervals (see SI Methods). The y axis corresponds to individual scores of the ASC-11D subscale ‘Spiritual Experience’.

SI METHODS

**Participants and Experimental Procedures**

Participants laid in the scanner with their eyes closed (an eye mask was used to prevent eyes opening), while brain activity and ECG were recorded. Following the scanning procedure, participants were interviewed and completed questionnaires designed to assess the subjective effects experienced during the scan. A second session then followed with the same procedure as the initial session, except on this occasion, participants were (audio) cued to verbally rate the subjective intensity of drug effects every minute in real time while in the scanner. This article reports the results concerning the ECG data collected during resting-state scans where participants received either DMT or a placebo, and were not interrupted for experience sampling purposes. Intensity scores were used for visualization purpose only (Figure 2), but were not included in the data analysis.

**Measures of ANS activity**

Each parameter value was first compared to their normal population values as presented in Nunan et al. (Nunan et al., 2010). The parameter values are then scaled with the standard deviations of normal population and a proprietary weighting is applied to obtain SNS and PNS indexes.

*Measure of sympathetic activity*

A Sympathetic Nervous System (SNS) index was calculated in Kubios using mean HR (higher heart rate is linked to higher sympathetic cardiac activation), Baevsky’s stress index (a geometric measure of HRV reflecting cardiovascular system stress, and sympathetic cardiac activation) and standard deviation along the line of identity of the R-R Pointcaré plot (SD2, see below) (Tarvainen et al., 2014).

*Measure of parasympathetic activity*

A Parasympathetic Nervous System (PNS) index was calculated in Kubios based on mean R-peak intervals (RR) (longer mean RR interval means lower heart rate and higher parasympathetic cardiac activation), the root mean square of the successive differences (RMSSD) - which is a commonly used time-domain HRV parameter that captures the quick beat-to-beat changes in RR interval, and therefore, strongly linked to respiratory sinus arrhythmia, a well-known measure of parasympathetic activity - and Poincaré plot index SD1 (Tarvainen et al., 2014).

*Measures of sympatho-vagal balance*

A measure derived from the PoinCaré plot of R-R intervals has been proposed as an index of sympatho-vagal balance. The Poincaré plot is a scatter plot of *RR_n_* vs. *RR_n+1_* where *RR_n_* is the time between two successive R peaks and *RR_n+1_* is the time between the next two successive R peaks. When the plot is adjusted by the ellipse-fitting technique, the analysis provides three indices: the standard deviation of instantaneous beat-to-beat interval variability (SD1), the continuous long-term R/R interval variability (SD2), and the SD1/SD2 ratio (SD12) (Bhaskar and Ghatak, 2013). SD1 indicates the dispersion along the minor axis of the Poincaré plot’s fitted ellipse. It reflects short-term HRV (instantaneous beat-to-beat variability) and correlates with baroreflex sensitivity and is therefore mainly influenced by parasympathetic modulation. SD2 indicates the dispersion along the major axis of the Poincaré plot’s fitted ellipse and reflects long-term RR interval fluctuations. SD2 increases suggests activation of both parasympathetic and sympathetic nervous system. SD1/SD2, which measures the unpredictability of the RR time series, is used to measure autonomic balance (Shaffer and Ginsberg, 2017).

**References**

Bhaskar R and Ghatak S (2013) Nonlinear Methods to Assess Changes in Heart Rate Variability in Type 2 Diabetic Patients. *Arquivos Brasileiros de Cardiologia* 101(4): 317–327.

Nunan D, Sandercock GRH and Brodie DA (2010) A quantitative systematic review of normal values for short-term heart rate variability in healthy adults. *Pacing and clinical electrophysiology: PACE* 33(11): 1407–1417.

Shaffer F and Ginsberg JP (2017) An Overview of Heart Rate Variability Metrics and Norms. *Frontiers in Public Health* 5: 258.

Tarvainen MP, Niskanen J-P, Lipponen JA, et al. (2014) Kubios HRV--heart rate variability analysis software. *Computer Methods and Programs in Biomedicine* 113(1): 210–220.
